# Complementary and Integrative Health Approaches Are Underused Among Older Veterans With Musculoskeletal Pain

**DOI:** 10.1093/geroni/igaa057.657

**Published:** 2020-12-16

**Authors:** Ling Han, Robert Kerns, Melissa Skanderson, Stephen Luther, Samah Fodeh, Joseph Goulet, Cynthia Brandt

**Affiliations:** 1 Yale School of Medicine, New Haven, Connecticut, United States; 2 VA Connecticut Healthcare System, West Haven, Connecticut, United States; 3 VA Connecticut Healthcare System, West Haven, CT, West Haven, Connecticut, United States; 4 James A. Haley Veterans Hospital, Tampa, Florida, United States; 5 Yale School of Public Health, West Haven, Connecticut, United States; 6 Yale School of Public Health, west haven, Connecticut, United States

## Abstract

Complementary and integrative health (CIH) approaches are recommended in national policy guidelines as viable options for managing chronic pain, yet their use among Veterans has been suboptimal, especially for older Veterans. We identified 64,444 Veterans with a diagnosis of musculoskeletal disorders (MSD) who reported a moderate to severe pain intensity during primary care visits in 2013 from the Veterans Health Administration (VHA) electronic records. Using natural language processing (NLP), CIH use (acupuncture, chiropractic care and massage) was documented for 8169 (6.5%) of 125408 primary care visits in providers’ progress notes. Compared to their younger counterparts, older Veterans aged ≥ 65 years had 21% lower likelihood of using CIH during the year [Odds Ratio (OR): 0.79; 95% Confidence Intervals (CI): 0.73, 0.86] after accounting for demographic, clinical, temporal and spatial confounding using a generalized estimating equation logistic model. Non-white race/ethnicity, tobacco use, medical comorbidities and diagnosis of alcohol or substance use disorders were independently associated with less CIH use (ORs ranging 0.97-0.80, p<0.03-0.0001); whereas female gender, being married and number of MSD diagnoses were associated with greater CIH use (ORs ranging 1.13-1.30, p<0.0001). Redefining CIH use as chiropractic care alone [4.8% person-visits; OR: 0.78 (95% CI: 0.70, 0.86)] or incorporating structured data [9.0% person-visits; OR: 0.76 (95% CI: 0.70-0.82)] in the adjusted GEE model derived consistent results. Research to identify and address barriers to CIH use among older Veterans is encouraged.

